# Physical Functioning, Self-Efficacy, and Quality of Life Among Older Cancer Survivors

**DOI:** 10.1093/geroni/igaf122.760

**Published:** 2025-12-31

**Authors:** Jessica Krok-Schoen, Cara Chase, Colleen Spees, Ashley Rosko, Mike Beck, Brett Nickerson, Roberto Benzo, Zachary Chaplow

**Affiliations:** The Ohio State University, Columbus, Ohio, United States; The Ohio State University, Columbus, Ohio, United States; The Ohio State University, Columbus, Ohio, United States; The Ohio State University Comprehensive Cancer Center, Columbus, Ohio, United States; The Ohio State University, Columbus, Ohio, United States; The Ohio State University, Columbus, Ohio, United States; The Ohio State University, Columbus, Ohio, United States; The Ohio State University, Columbus, Ohio, United States

## Abstract

**Introduction:**

Older cancer survivors are at heightened risk of physical limitations, which can negatively impact their quality of life and self-efficacy for healthy behaviors. The E-PROOF (E-intervention for Protein Intake and Resistance Training to Optimize Function) study is the first synchronous, online, protein-focused dietary and resistance training randomized controlled trial among older cancer survivors.

**Objective:**

This study seeks to understand the changes in and association among physical functioning, self-efficacy, and quality of life among older cancer survivors over the 12-week online diet and resistance training intervention.

**Methods:**

Eligibility criteria include adults age ≥65 years, stage I-III breast, colorectal, or prostate cancer, and completion of curative treatment. Intervention participants received online nutrition counseling sessions and online resistance training sessions. Control participants received online counseling on stretching and healthy eating.

**Results:**

In interim analysis (n = 61, median age=70), 72% of the sample are breast cancer survivors. Improvements in physical function were observed for the whole sample (mean scores=9.6 and 10.2, p = 0.054). Participants also reported improvements in exercise self-efficacy and dietary self-efficacy. Health-related quality of life significantly improved in physical function (75.3 to 81.7, p = 0.03), physical role limitations (55.7 to 73.4, p = 0.01), and energy (58.7 to 69.3, p = 0.001). No significant differences were observed between groups. Significant positive correlations were observed after the intervention among physical function, quality of life, and self-efficacy. Final sample results will be presented.

**Conclusions:**

Trial results will inform the preliminary efficacy of online diet and resistance training interventions to improve physical function and related psychological outcomes among older cancer survivors.

